# Impact of the First COVID-19 Lockdown on Management of Pet Dogs in the UK

**DOI:** 10.3390/ani11010005

**Published:** 2020-12-22

**Authors:** Robert M. Christley, Jane K. Murray, Katharine L. Anderson, Emma L. Buckland, Rachel A. Casey, Naomi D. Harvey, Lauren Harris, Katrina E. Holland, Kirsten M. McMillan, Rebecca Mead, Sara C. Owczarczak-Garstecka, Melissa M. Upjohn

**Affiliations:** Dogs Trust, London EC1V 7RQ, UK; katharine.anderson@dogstrust.org.uk (K.L.A.); Emma.Buckland@dogstrust.org.uk (E.L.B.); rachel.casey@dogstrust.org.uk (R.A.C.); naomi.harvey@dogstrust.org.uk (N.D.H.); lauren.harris@dogstrust.org.uk (L.H.); Katrina.Holland@dogstrust.org.uk (K.E.H.); kirsten.mcmillan@dogstrust.org.uk (K.M.M.); rebecca.mead@dogstrust.org.uk (R.M.); Sara.Owczarczak-Garstecka@dogstrust.org.uk (S.C.O.-G.); melissa.upjohn@dogstrust.org.uk (M.M.U.)

**Keywords:** dogs, COVID-19, lockdown, behaviour, welfare, human–animal interaction, management

## Abstract

**Simple Summary:**

Initial COVID-19 lockdown restrictions in the United Kingdom (23 March–12 May 2020) prompted many people to change their lifestyle. We explored the impact of this lockdown phase on pet dog welfare using an online survey of 6004 dog owners, who provided information including dog management data for the 7 days prior to survey completion (4–12 May 2020), and for February 2020 (pre-lockdown). Most owners believed that their dog’s routine had changed due to lockdown restrictions. Many dogs were left alone less frequently and for less time during lockdown and were spending more time with household adults and children. During lockdown, dogs were typically walked less often and for less time daily, with factors related to the dog, owner, household, and location of the home associated with the extent to which dog walking had been reduced. Dogs were more likely to be walked on a lead and had fewer opportunities to interact with other dogs during lockdown. However, many dogs had more play/training sessions with their owners and were given toys more frequently during lockdown. These changes to dog management have the potential for longer-term welfare problems such as increased likelihood of dogs displaying separation-related behaviour as lockdown measures relax.

**Abstract:**

Initial COVID-19 lockdown restrictions in the United Kingdom (23 March–12 May 2020) prompted lifestyle changes for many people. We explored the impact of this lockdown phase on pet dogs using an online survey completed by 6004 dog owners, who provided information including dog management data for the 7 days prior to survey completion (4–12 May 2020), and for February 2020 (pre-lockdown). We explored associations between potential predictors and four outcomes relating to changes pre-/during lockdown (reduction in number and duration of walks; increased frequency of play/training, and provision of toys). Most owners (79.5%) reported their dog’s routine had changed compared to pre-lockdown. There was a four-fold increase in the proportion not left alone for >5 min on any day during a weekly period (14.6% pre-lockdown, 58.0% during lockdown), with the proportion being left for ≥3 h at a time decreasing from 48.5% to 5.4%. Dogs were walked less often and for less time daily during lockdown, with factors related to the dog, owner, household, and home location associated with changes to walking practices. Many dogs had more play/training sessions and were given toys more frequently during lockdown. Decreased walk duration was associated with increased odds of play/training opportunities and toy provision. These changes to dog management have the potential for immediate and longer-term welfare problems.

## 1. Introduction

A novel coronavirus, SARS-CoV-2, linked to cases of human viral pneumonia was first reported in Wuhan, People’s Republic of China in December 2019, and the disease it causes was subsequently named COVID-19 in February 2020 [1]. The first case of SARS-CoV-2 transmission inside the United Kingdom (UK) was confirmed on 28 February 2020 [2]. The World Health Organisation (WHO) characterised COVID-19 as a pandemic on 11 March 2020 [3] and called for countries to take urgent and aggressive action to prevent infections. Restrictions were imposed by many countries, including the UK, with the aim of reducing transmission of SARS-CoV-2 and reducing the basic reproduction number of the virus (R_0_). Such restrictions have been colloquially referred to as “lockdown” restrictions.

Lockdown restrictions around the message of “stay at home” were announced by the UK government on 23 March 2020 [4], restricting the operation of business and leisure activities, thus leading many people to adopt changes in lifestyle. On 13 May 2020, some UK lockdown restrictions were eased with differences in restrictions occurring across the four devolved nations of the UK (England, Wales, Scotland, and Northern Ireland).

Restrictions across the UK (in place 23 March–12 May 2020), and associated changes in lifestyle, were expected to have had an enormous effect on the lives of many people. The UK Coronavirus Job Retention Scheme designed to reduce the risk of redundancy led to 6.3 million jobs being furloughed by 3 May 2020 [5], with many of those furloughed receiving less income and experiencing potentially increased job insecurity, whilst increasing the time that furloughed staff would be spending at home. People that were still working were more likely to work from home compared to pre-pandemic arrangements—see comparative data from 2014–2015 [6] and a recent study which was undertaken during 2020 [7]. The latter described the proportion of survey respondents who reported that they worked from home “always” or “often” as having increased from 13.2% to 43.7% based upon 9931 respondents who reported the extent to which they worked from home during January/February 2020 and at the point of survey completion during lockdown (23 April–1 June 2020) [7]. The composition of some households also changed as a result of decisions made when lockdown regulations were announced, with friends/relatives from different households living together to reduce social isolation and to provide assistance to the elderly/vulnerable [8] whilst others began living apart to reduce exposure risk to vulnerable family members from those considered “key workers” [9].

Although regulations varied between countries, documented consequences for Spanish householders subject to lockdown regulations included lifestyle, emotional, financial, and health impacts [10], with lifestyle impacts reported to negatively affect 49.2% of survey respondents. Anticipated implications specific to dog owners worldwide, including the UK, relate to changes affecting the human–animal bond, emotional support provided by the dog during the pandemic, and anxiety around aspects of caring for the dog, for example restrictions to nonessential veterinary care such as in the UK [11]. Pet dogs living in households were also potentially affected, in terms of their management, behaviour, and the human–animal bond. This paper focuses on owner-reported management of dogs and changes to these practices that occurred during lockdown, which were hypothesised to have the potential to impact the short- and/or longer-term welfare of pet dogs within UK households [12]. Changes in management of dogs were anticipated to occur due to people being instructed to stay at home (with a few, specified exceptions including allowing those defined as “key workers” [13] to continue working); increased time spent at home, resulting in part from furloughing and increased working from home practices; closure of schools; outdoor exercise away from the home being limited to once a day per individual. There is potential for short-term changes that might occur during lockdown (e.g., reduced exercise, reduced social contact with dogs/people whilst out on walks, reduced time that dogs were left alone without human contact, and less opportunity for dogs to have “quiet time” away from people) to lead to longer-term canine welfare problems (e.g., increased reactivity to dogs and/or people, increased separation-related behaviour (SRB)). These could impact the canine welfare sector and the UK pet dog population beyond the initial lockdown period. 

In Spain, restrictions in place 14 March–2 May 2020 specified that dogs were allowed to be walked on the lead by one person who should observe social distancing rules, but that walks should be kept to an absolute minimum, i.e., of short duration to permit toileting [14,15]. Following an average length of confinement of 3.2 weeks, 62.1% of 794 dog owners surveyed within Spain considered that their dog’s overall quality of life had worsened during the period of lockdown confinement, whilst 19.3% of owners thought their dog’s overall quality of life had improved [10]. When asked about the occurrence of behaviours often considered problematic, many Spanish dog owners perceived their dog’s behaviour to have “got worse”, with specific increases in problems with being left alone at home (11.8%), annoying or excessive vocalisation (24.7%), attention-seeking behaviour (41.6%), nervous behaviour (24.9%), and excitable behaviour (20.8%). However, 29.5% of surveyed dog owners in Spain did not detect any significant changes in their dog’s behaviour whilst restrictions were in place [10]. 

This paper describes data collected from online survey completion by UK dog owners between 4 and 12 May 2020, i.e., during the first “strict” phase of the COVID-19 lockdown in the UK, and before widespread initial easing of lockdown restrictions.

The aim of the paper is to describe and explore changes in dog management practices resulting from the implementation of the COVID-19 lockdown restrictions compared with a pre-lockdown period (early/mid-February 2020) in order to identify potential short- and longer-term impacts of this event on UK pet dogs. Specifically, we focus on changes to three areas we hypothesise to have been substantially impacted during lockdown: dog walking practices, interactions with people and other dogs, and enrichment activities. Longer-term impact(s) of the COVID-19 lockdown are being quantified in ongoing separate analyses of data collected longitudinally from the dog owners who provided data presented in this paper. 

## 2. Materials and Methods 

A cross-sectional study design was used to investigate factors related to dog acquisition/relinquishment, owner sentiments, and the management and behaviour of pet dogs during the initial UK “lockdown” phase of the COVID-19 pandemic between study launch (4 May 2020) and 12 May 2020. This paper focuses on data regarding dog management practices. Dog owners were asked for consent to be contacted for completion of a follow-up survey, so that long-term impacts on canine health and behaviour related to lockdown can be assessed in the future.

### 2.1. Survey Tool 

Data were collected using a self-administered online survey created using SmartSurvey^TM^ software (https://www.smartsurvey.co.uk/). The survey was piloted by members of the Dogs Trust research team and other Dogs Trust staff members who owned dogs and refined before the final version was finalised [16]. Questions included dog and owner demographic information, owner reports of dog behaviour, management/environment of the dog and data relating to household composition. Many questions required owners to describe the dog’s behaviour/management during the last 7 days (i.e., during the first phase of lockdown), and also during early/mid-February 2020 (i.e., before social distancing measures likely to lead to changes in owners’ behaviour had been introduced). The majority of questions were optional.

Most questions presented respondents with the option to select one or more predefined responses, sometimes with the option to add in free text “other” responses if required. The survey was estimated to take approximately 25 min to complete. This paper focuses on results from three specific areas of interest, i.e., changes to dog walking practices; interactions with people and other dogs; enrichment activities. In terms of dog walking, we focused on the number and duration of walks per day, choice of walk location, the use of the lead (including use of short and long/flexi-leads), and off lead walking practices (to heel and not to heel). Analysis of interactions with people and other dogs included evaluation of changes to the people and dogs lived and interacted with, opportunities to interact with other dogs (including sniffing and playing with other dogs while on walks), and the amount of time dogs were left alone. Changes to enrichment practices that included and excluded direct interaction with people were also evaluated, focusing on training and play, and provision of toys, respectively.

### 2.2. Inclusion Criteria 

Study participants were required to be at least 18 years of age, to live in the UK and to either currently own a dog, or to have owned a dog when the COVID-19 lockdown restrictions were introduced on 23 March 2020. Owners were instructed to complete the survey for the dog that had joined the household most recently, and if more than one had joined at the same time, then the dog whose name was first alphabetically (the “participating dog”). The dataset was screened, and duplicate responses (based on IP address) were removed, retaining the survey with the least amount of missing data. 

### 2.3. Recruitment 

The survey URL was live from 4 May 2020 and advertising of the survey commenced on 5 May 2020. The survey was advertised through social media including paid Facebook advertisements, a Dogs Trust e-newsletter, New Scientist magazine, and emails to dog owners participating in the Generation Pup study (www.generationpup.ac.uk) or the Dogs Trust Post Adoption study who gave consent to be contacted this way. Participants of a previous survey administered by the Dogs Trust research team who had consented to be contacted about further research opportunities were also invited to participate.

Data presented here were collected between 4 and 12 May 2020, after which government guidelines around exercise limitations began to be relaxed across the four nations. 

### 2.4. Data Analysis

Initial data analysis included calculation of descriptive statistics (frequency and percentage) for all survey results, including demographic and household characteristics for both owner and dog, at the two timepoints of interest: pre-lockdown (early/mid-February 2020) and during lockdown (“the last 7 days”). For simple comparisons between the time points, and as data were paired (variables reported for pre-lockdown and lockdown), McNemar’s tests were used to test for associations between binary categorical variables and Stuart–Maxwell tests for ordinal and nominal variables. Generalised linear models (GLM) with logit link functions were used to test for associations between potential predictor variables and four binary outcomes of interest: reduction in the number of walks per day during lockdown, compared with pre-lockdown; reduction in the total duration of walks per day during lockdown, compared with pre-lockdown; increased frequency of providing toys to the dog during lockdown, compared with pre-lockdown; increased frequency of play/training with dog during lockdown, compared with pre-lockdown. Owner-reported frequency and duration of dog walks was used to calculate two binary variables indicating whether the frequency and duration of their dog’s walks had decreased or not. For both of these variables, 1 was recorded where the frequency/duration had decreased, regardless of magnitude (e.g., from “once per day” to “not walked”, or from “2+ h per day” to “<30 min per day”), and 0 for no change or an increase in frequency/duration. All analyses assessing change from before to during lockdown only included participants who indicated that they acquired the dog prior to the end of January 2020 and provided data for both time points. Hence these analyses excluded, for example, people who acquired their dogs during lockdown.

Similar to walks, owner-reported frequency of toy provision and play/training with the dog was used to create two binary variables indicating whether the frequency with which dogs were provided with a toy (i.e., an opportunity for enrichment was provided that did not involve sustained dog–human interaction) or were engaged in play or training (i.e., interactions involving the dog and a person). For both these variables, 1 was recorded where the frequency increased, regardless of the magnitude (e.g., from ’less than once a week’ to ‘once a day’), and 0 for no change or a reduction in frequency. The associations between these variables and selected other variables were explored using multivariable logistic regression to identify potential risk factors for increased toy provision and play/training.

In each case, initial multivariable models included respondent age group, respondent gender, number of dogs in household, age category of dog, sex of dog, area of residence (rural, city etc.), access to off lead walking (directly from the house, after walking on lead, only by car/public transport), number of adults currently in house, presence/absence of children, whether or not home has a garden/yard or communal garden, and whether or not an additional person was now walking the dog. Further information about these variables is available in the Supplementary Information. In addition, walk frequency and walk duration were considered for inclusion in the multivariable models for increased frequency of play/training and toy provision. However, both variables (walk frequency and walk duration) could not be included in the same model due to the correspondence of the category “Not walked”. Hence, two models were created for each outcome variable (increase in play/training and provision of toys), one with the variable of walk frequency and one with the variable of walk duration. Subsequently, nonsignificant variables were removed sequentially from the models, beginning with the nonsignificant variable with the highest *p*-value. Variables were retained in the model if they improved the fit of the model (likelihood ratio test *p*-value ≤ 0.05). Models based on the same number of participants were always used when nested models were compared. Plausible interactions were also tested and retained if significant. All data handling and analysis was conducted using R v4.0.1 [17] via RStudio^®^ v1.3.959 [18]. 

### 2.5. Ethics 

The study was approved by the Dogs Trust Ethical Review Board (ERB036). 

## 3. Results

Between 4 and 12 May 2020, a total of 6505 surveys were submitted online by dog owners. Following data cleaning, including removing duplicate responses, 6004 surveys were available for analysis. Over three-quarters of respondents (76.7%, *n* = 4604/6004) reported that they obtained the participating dog before the end of January 2020, 4.4% (*n* = 267) had obtained their dog after January 2020, but before lockdown began on 23 March 2020, and 1.0% (59) had obtained their dog since 23 March 2020 (1%, *n* = 58 answered “do not know/cannot remember” or “prefer not to say”, and 16.9%, *n* = 1016 responses were missing). Results presented in this paper only included participants who indicated that they acquired the dog prior to the end of January 2020 (*n* = 4604). Most questions were optional, so reported percentages are based on available responses to individual questions, which frequently numbered <4604.

### 3.1. Owner Demographics and Household Characteristics

The vast majority (85.7%, *n* = 3429/4000) of respondents were female, 14.2% (*n* = 566) were male and 0.1% (*n* = 4) self-identified). Around two-thirds of respondents (66.1%, *n* = 3041/4598) reported that they owned one dog, almost a quarter (22.3%, *n* = 1068) reported owning two, 6.7% (*n* = 310) owned three dogs, and 2.0% (*n* = 94) owned four dogs. Just 1.8% (*n* = 85) owned five or more dogs, including three respondents that reported owning more than 10 dogs. 

Over half of the participating dogs lived with two adults (58.0%, *n* = 2336/4028), 21.7% (*n* = 875) with one adult, 13.2% with three adults (*n* = 530), and 5.6% (*n* = 225) with four adults. A few dogs lived with five (1.4%, *n* = 55) or a maximum of six adults (0.2%, *n* = 7). Most dogs did not live with children (84.3%, 3387/4016). Of those dogs living with children, 57.1% (359/629) lived with one child, 36.7% (*n* = 231) with two, 4.9% (*n* = 31) with three, 1.1% (*n* = 7) with four, and 0.2% (*n* = 1) with 10.

Some households (9.2%, *n* = 357/3893) included adults living with them during lockdown who were not normally resident in the household. In most cases (6.3%, *n* = 246/3893) this was a single additional adult, while 2.3% (*n* = 88) had two, 0.4% (*n* = 16) had three, 0.1% (*n* = 5) had four, and 0.1% (*n* = 2) had six extra adults. Similarly, some households (4.5%, *n* = 173/3846) had adults absent that were usually resident. In these cases, usually there was one (3.7%, *n* = 143/3846) or two (0.7%, *n* = 26) adults absent, also a few (0.05%, *n* = 2 in both cases) had three or four absent. In total, 9.5% (*n* = 374/3919) households had one or more additional adults and/or one or more absent adults compared with pre-lockdown. Details regarding the respondent’s age category, the number of dogs in their household and the age category of the dog were also summarised (see Table S1).

The vast majority of respondents (81.3%, *n* = 3569/4390) reported that at the time of survey completion they only left their house for one or more of the reasons named as essential by the Government (including shopping, medical appointments, exercise including dog walking, caring for vulnerable people). Some (14.8%, *n* = 650) reported leaving their house for work, while a small percentage (3.9%, *n* = 171) reported that they were self-isolating and did not leave the house/garden at all. Somewhat similar patterns were evident for other adults in the households (see Table S2). 

The majority of respondents lived in a village or small town (41.1%, *n* = 1648/4010) or in a suburban area (28.3%, *n* = 1133). About one in eight (13.3%, *n* = 535) lived in city or urban areas and 17.3% (*n* = 694) lived in rural (16.0%, *n* = 642) or remote (1.3%, *n* = 52) areas. Almost all dogs (95.9%, *n* = 3737/3896) were reported to have access to a private garden or yard, with a few having access to a communal garden or yard (2.4%, *n* = 93). Less than 2% did not have access to a garden or yard (1.7%, *n* = 66). In most cases, gardens and yards were enclosed and dog-proof (90.8%, *n* = 3652/4021), whereas some were enclosed but not dog proof (5.5%, *n* = 221), open to the street (1.0%, *n* = 42), or open to the countryside (2.6%, *n* = 106). Around two-thirds of respondents (67.7%, *n* = 2711/4006) reported they could walk out of the house with their dog on a lead to access off lead areas for exercise, whilst 10.0% (*n* = 401) could access off lead exercise areas directly from their house. Some (7.8%, *n* = 314) needed (private or public) transport to access off lead exercise areas and 14.5% (*n* = 580) were not walking their dog off lead pre-lockdown.

### 3.2. Dog Demographics

**Figure 1:**
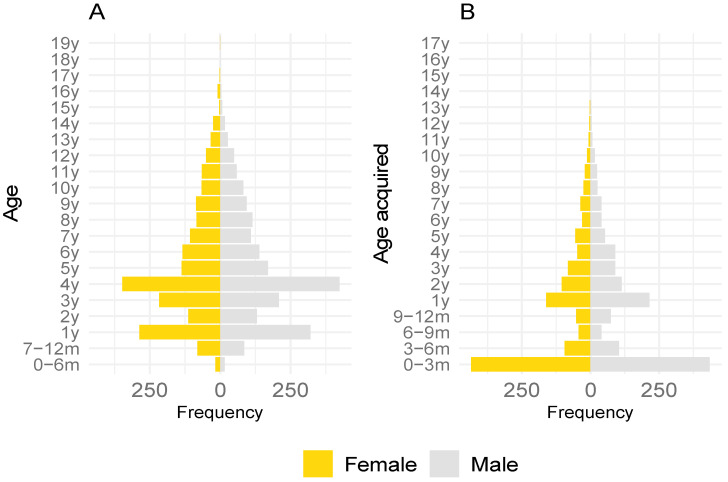
Age (**A**) and age acquired (**B**) population pyramids for participating dogs. Note, in general category labels range from greater the value given to less than or equal to the subsequent value, so “1y” indicates an age >1 year and ≤2 years, likewise “3–6 m” indicates an age >3 months and <6 months.

For dogs where breed was reported (99.8%, *n* = 4597/4604), the majority were a specific breed (55.6%, *n* = 2557/4597), whereas 27.2% were a mix of two specific breeds (*n* = 1252) and 17.1% were other or unknown breeds or mixes (*n* = 788). Respondents reported data for 2156 female and 2422 male dogs (47.1% females and 52.9% males of 4758 dogs whose owners provided information on the sex of their dog). The mean age of dogs was 5.1 years (standard deviation (SD) = 3.6 years, minimum 0.25 years (90 days), 1st quartile 2.2 years, median 4.1 years, 3rd quartile 7.4 years, maximum, 19.4 years, with age not provided for 659 dogs). The mean age of male dogs was 5.1 years (SD = 3.5) and for females was 5.2 years (SD = 3.7). The most common age groups were 4.0 years and 1.0 years (Figure 1).

The distribution of age at which dogs were acquired was highly skewed (Figure 1), with mean 2.3 years old (SD = 2.0 years, minimum = 0 years (36 people reported this, presumably having bred the dog themselves), 1st quartile 0.21 years, median 1.1 years, 3rd quartile 3.5 years, maximum 16.5 years, with age acquired not provided for 1999 dogs). Almost half the participating dogs (for which these data were available) were acquired before 1 year of age (49.3%, *n* = 1283/2605), with most of these acquired by 3 months of age (33.5%, *n* = 872/2605). Around one in seven dogs (14.5%, *n* = 377/2605) were acquired as 1-year-olds and the frequency of acquisition declined as age increased.

### 3.3. Changes in Daily Routine

More than three-quarters of respondents reported that their dog’s daily routine had changed: 56.8% (*n* = 1283/4120) “a little” and 22.9% (*n* = 942) “a lot” at the time of survey completion, compared to pre-lockdown in February 2020. However, around one in five reported no change in their dog’s routine (20.3%, *n* = 836).

### 3.4. Changes in Dog–Human Interactions

While most dogs (89.6%, *n* = 4312/4595) were still being walked by the same person or people as in the period immediately prior to lockdown, some owners reported that their dogs were (also or solely) being walked by other familiar (6.6%, *n* = 319/4595) or unfamiliar (1.0%, *n* = 46/4595) people who did not walk the dog in the period prior to lockdown. Some dogs had not been walked in the previous 7 days (2.0%, *n* = 96) or in the period prior to lockdown (0.8%, *n* = 38).

Dogs were reported to be spending more time with human members of their household (Table 1). Nearly 70% of dogs were spending more time with adults (20.5% “a little more” and 49.3% “much more”) and over 86% were spending more time with children (15.1% “a little more” and 71.3% “much more”).

### 3.5. Changes in the Time That the Dog Is Left Home Alone

As anticipated, in comparison to pre-lockdown the extent to which dogs were left alone greatly decreased during lockdown, both in terms of frequency and maximum duration (Table 2). The proportion of dogs that were not left at home (for at least 5 min) on any day during a weekly period increased four-fold, from 14.5% prior to lockdown to 57.8% during lockdown. The proportion of dogs left for 3 or more hours at a time reduced from 48.4% prior to lockdown to only 5.4% during lockdown.

### 3.6. Changes in Dog Walking Practices: Frequency and Duration

During the initial phase of lockdown investigated here, just over half of dogs had reportedly been walked once or less per day during the last 7 days, 36.2% were walked twice a day and 11.8% were walked three or more times a day (Table 3). This represented a significant change to the pattern prior to lockdown, with an increase in the proportion of dogs walked once per day, and a decrease in the proportion of dogs walked 2–4 times a day. Although the proportion of dogs not walked at all more than doubled, this still only impacted a small proportion of the population (2.4% compared to 1.0% prior to lockdown).

Despite a significant reduction in the number of walks for many dogs, the reduction in walk duration from prior to during lockdown, while still statistically significant, was less marked. Most dogs (79.3%) were reportedly walked between 30 min and 2 h per day during the previous 7-day period of the early phase of lockdown, compared with 77.9% of dogs prior to lockdown. The main difference between the study periods was in the number of dogs not walked at all, although again this affected only a small number of dogs (the number of dogs reported as not being walked varied slightly for the questions about frequency and duration of walking, predominantly due to some people failing to answer this question for duration).

### 3.7. Changes in the Number of Dogs “Met” on an Average Day

The number of dogs (excluding other household dogs) that owners’ dogs had “met” (i.e., been in the same room, or within 2 m if outside) on an average day decreased markedly. Prior to lockdown, 8.6% of dogs met no other dogs on an average day, which increased three-fold to 26.3% of dogs during lockdown (Table 3).

### 3.8. Choice of Walk Location, Use of Short/Long Leads, and Interactions with Other Dogs

The choice of walk location based on the likelihood of meeting other dogs (and therefore people) differed markedly between before and during lockdown (Table 4). A little over half of owners chose their walk location based on reasons other than the likelihood of meeting other dogs at each time point. Among the remainder of owners there was an increase in the proportion seeking to avoid walking in places where there were likely to be other dogs during lockdown (34.6% compared with 21.5% pre-lockdown). These choices were reflected in the areas where dogs were usually walked during lockdown. These areas were more likely to have no or few dogs and, where areas were visited by other dogs, these were more often reported to be walked on lead when compared with prior to lockdown. This pattern was observed despite walking locations being less likely to be rural during lockdown (perhaps due to reduction in driving for exercise) (Table 5). This increased tendency for other dogs to be on lead reflected our respondents’ own behaviour, in that they were more likely to walk their dogs on a short lead or on a long/flexi-lead and less likely to walk their dogs off lead not to heel during lockdown compared to before lockdown (Table 4).

In addition to these changes to reduce encounters with other dogs while walking, during lockdown owners were also more likely to prevent their dog interacting (i.e., sniff and/or play) when other dogs were observed, regardless of whether the other dog was familiar or not, or if their dog was on a lead or not (Table 5). For example, if the owner’s dog was on the lead when they met an unfamiliar dog, prior to lockdown 56.9% of dogs would have been allowed to meet the other dog compared with 30.2% during lockdown.

The increased tendency for owners to avoid walking their dogs in places where other dogs were likely to be, to use leads to a greater extent, and to limit their dog’s ability to interact with other dogs were factors likely to have contributed to the reduction in time dogs were reported to spend interacting with other dogs (Table 6). Compared to prior to lockdown, during lockdown the majority of dogs spent less time in social contact with dogs from outside the household (43.3% much less and 16.0% a little less time); with few (6.8%) spending more time interacting with other dogs.

Reduction in the daily frequency of walking was associated with variables related to the respondent and their location, the dog, and the household. Compared to rural/remote areas, respondents in each of the other areas (from village to city) were significantly more likely to report a decrease in walk frequency (Table 7; model 1). Even after controlling for area, people who could walk their dog off lead directly from their home were significantly less likely to report reduced frequency of dog walking. Owners of younger dogs (i.e., those age groups from 7 months to under 2 years of age), were significantly more likely to report reduced walk frequency than senior adult dogs (7–11 years). Walk frequency was not different for puppies (≤6 months) or for older age categories (mature adult, 2–6 years; geriatric, 12 years or more) when compared to walk frequency reported for senior adult dogs (7–11 years). Household membership, and the “use” of additional dog walkers also impacted frequency of a dog’s walks. Households with more than one adult were significantly less likely to report a reduction in walk frequency, and this followed a gradient with greater reduction in odds as the number of adults increased. In addition, the help of one or more additional adults that had not walked the dog prior to lockdown also made it less likely that a dog’s walk frequency was decreased.

Three of the variables associated with frequency of dog walks were also associated with the odds of reduction in daily duration of walks, with somewhat similar effect (Table 7; model 2). Compared to those living in rural/remote locations, other respondents were more likely to report a decreased duration of dog walks. Ease of access to off lead exercise areas also affected duration of walking, with those requiring transport to access off lead exercise areas having greater odds of reporting shorter walk duration. Households with more adults also had lower odds of decreased walk duration, particularly with four or more adults.

### 3.9. Changes in the Frequency of Enrichment Practices Such as Provision of Toys, as Well as Playing and Training with People

There was evidence of a change in the frequency with which dogs were given toys to play with from before to during lockdown (Table 8). Overall, (excluding people who reported they could not remember for either time point) 86.3% (*n* = 3504/4059) did not change the frequency with which they gave their dog a toy, while 9.0% (*n* = 367) reported an increased frequency and 4.6% (*n* = 188) a decreased frequency. Of those that increased the frequency with which they gave a toy, 65.1% (*n* = 239) did so by one category on the ordinal scale, 22.6% (*n* = 83) by 2, 9.3% (*n* = 34) by 3, 2.7% (*n* = 10) by 4, and one person who had not previously given a toy did so once a day. Where respondents indicated the frequency had decreased, 60.4% (*n* = 113) reported a 1 category decrease, 21.9% (*n* = 41) 2 categories, 11.2% (*n* = 21) 3, 4.8% (*n* = 9) 4, 0.5% (*n* = 1) 5, and three (1.6%) people who had not given a toy prior to lockdown, reported that they had done so during lockdown. This question referred to a wide range of toy-types, including toys with food inside (e.g., a stuffed Kong or snuffle mat), puzzle or “brain games”, tugger or rope toys, bones, chews or rawhide treats, ball, soft toys (e.g., teddies), any other toys (homemade or bought), and specifically indicated the frequency with which the dog was given these toys to play with on their own or with another dog. The frequency with which people played with or did some training with their dog increased during lockdown (Table 8), with the greatest increase seen in the percent of responses in the “more than once per day” category (38.3% pre-lockdown compared to 48.5% during lockdown). Nevertheless, excluding people who reported they could not remember for either time point, 70.2% (*n* = 2856/4069) of respondents reported they had not changed the frequency with which they engaged in play or training with their dog, while 23.2% (*n* = 946) reported an increased frequency (6.6%, *n* = 267, reported decreased frequency). Of those that increased the frequency of play and training, 62.4% (*n* = 590) did so by one category on the ordinal scale, 20.3% (*n* = 186) by 2, 13.5% (*n* = 128) by 3, 4.0% (*n* = 38) by 4, 0.3% (*n* = 3) by 5, and one person who had not previously played/trained reported doing so more than once per day by 5. Where respondents indicated the frequency had decreased, 60.7% (*n* = 162) reported a 1 category decrease, 20.2% (*n* = 54) 2 categories, 13.1% (*n* = 35) 3, 4.1% (*n* = 11) 4, 1.5% (*n* = 4) 5, and one person who had previously engaged in play/training did not do so during the week reported during lockdown.

Variables related to the respondent’s location, the respondent, the dog, and walking practices were associated with the odds of increased frequency of play/training (Table 9). In terms of walking practices, both walk frequency and walk duration were associated with the odds of increased frequency of play/training. The variable “change in walk frequency” was not retained within the multivariable model used to test this variable (data not shown), whereas the variable “change in walk duration” was associated with the odds of increased frequency of play/training in the multivariable model and is included in the final model summarised in Table 9. Owners who reported that their dog’s walking duration had changed (increased or decreased) had increased odds of reporting increased frequency of play/training, compared to those reporting no change in walk duration (based on the categories available for selection). People in suburban areas were most likely to report increased frequency of play/training (Odds Ratio 1.6 compared to the reference area rural/remote). Compared to respondents aged “45–54 years”, all younger age categories were significantly more likely to report an increased frequency of play/training, while older age categories were significantly less likely to report this. Female respondents were also significantly more likely to report increased play/training, as were households in which a new adult was involved in dog walking.

Three variables were associated with increased odds of providing toys for the dog to play with during lockdown (Table 9). Compared with male respondents, females had increased odds of increasing the provision of toys for their dogs to play with during lockdown. Compared to respondents aged “55–64 years”, younger age categories up to 44 years of age were significantly more likely to report an increased provision of toys, while the “45–54 years” category and older age categories were not significantly different. Those respondents reporting a decreased duration of walks were more likely to report an increased frequency of providing toys for their dogs, compared to those who reported no change in walk duration (based on the categorical responses available in the survey). The variable of “change in walk frequency” was not associated with the odds of increased frequency of providing toys in the final multivariable model.

## 4. Discussion

This study provides novel insights into the impact that the COVID-19 lockdown restrictions in the UK had on dogs and their owners, as a result of lifestyle changes that occurred during this period. Specifically, we hypothesised that impacts would include alteration to the daily routine of dogs, dog–dog and dog–human interactions (including time left alone), dog walking (frequency and duration), provision of enrichment (toys and play/training), and these are discussed further below.

We found that over three-quarters of respondents reported their dog’s daily routine had changed since the commencement of lockdown. Collectively, there are implications to changing dog’s routines, which could result in anxiety or frustration due to expecting or anticipating events which were no longer occurring [19]. For example, if a usual morning walk does not occur then the dog may become anxious in anticipation of the walk, and lack of the walk could lead to frustration and behaviours such as attention-seeking behaviour and/or whining or barking. Loss of predictability is often considered to be aversive, acting as a stressor for captive/managed animals, with negative impacts on animal welfare (see [19] for review). Specific routine changes can also lead to changes in physical exertion and/or mental stimulation which in themselves may have welfare consequences; however, we are not aware of any studies specifically investigating the impacts of changes to routine for household dogs. Conversely, some of the changes in routine might have resulted in improved dog welfare. Whilst this study did not aim to assess dog welfare, we focused on aspects which are hypothesised to impact on dog welfare such as time left alone, frequency and duration of dog walks, enrichment through the provision of toys, and interacting with the owner during games/training. Future research will include analyses to identify and quantify associations between management changes and owner-reported observations of dog behaviour (before, during and after lockdown) that can be considered to be indicative of dog welfare.

### 4.1. Changes in Dog Walking Practices

At the start of lockdown people in the UK were initially limited to one form of outdoor exercise per day with no additional exercise permitted for dog owners [20]. Social distancing requirements (to stay at least 2 m away from others) were also in place, potentially reducing off lead exercise and opportunities for dogs to interact with other people and dogs [21]. Some owners might have avoided interacting with dogs from outside their household if concerned that the virus could be transmitted to humans by touching dog hair, which had been suggested as a possible route of transmission [22,23] although no scientific evidence exists to support this theory [24]. Other owners might have avoided contact with dogs from outside their household to avoid virus transmission via fomites such as collars, harnesses, and leads. Our results indicated that the experience for dogs of being walked was changed in numerous ways during the lockdown, compared with our reference period of early/mid-February 2020 (i.e., before lockdown and prior to social distancing being recommended). Firstly, lockdown resulted in a marked reduction in the daily frequency of walks for many dogs and some reduction in total duration of walking. Although still relatively infrequent, the number of dogs not walked at all more than doubled during lockdown but remained broadly similar to previous estimates [25,26]. Lockdown appeared to result in a substantial increase in the number of dogs walked once per day, with the most marked reduction in the number of dogs walked three or more times per day—changes that were anticipated due to the government rule of one period of outdoor exercise per person per day. Although we found a significant change in the duration of dog walks, this was not as marked as for frequency, perhaps because owners chose to undertake fewer but longer walks, although it is also possible that the response categories available to record walk duration were insufficiently sensitive to more clearly detect changes in duration.

Our results may have underestimated the impact of exercise restrictions on frequency and duration of dog walking, if reduced daylight length and less favourable weather conditions in February naturally suppressed dog walking compared to the more favourable walking conditions of early-mid May 2020 [27], although there is limited research of the effect of weather conditions/season on frequency/duration of dog walking [28]. Comparative data on dog walking frequency prior to the COVID-19 pandemic (with little missing data that would bias the reported estimates [29]) revealed 32.6% of dogs were reportedly walked twice per day and 15.6% were walked more than twice per day [25]. In addition, it has previously (pre-2020) been estimated that 13–22% of dogs living in the UK might not be walked every day [25,26]. We acknowledge that our dataset is likely to have over-representation of dog owners committed to canine welfare, as indicated by their willingness to complete a 25-min online survey about dog ownership. Consequently, dogs within our sample might have been walked more frequently (at baseline) than the wider UK pet dog population [25,26], and a greater decline in exercise frequency and duration might be anticipated for dogs owned by people less able and/or willing to meet the physical and mental needs of their dogs. Nevertheless, for dogs receiving infrequent (less than daily) walks at baseline, it is likely that lockdown had less impact in terms of reducing walk frequency than for dogs that were walked more frequently.

Several factors were found to be associated with reduced dog walking frequency and/or duration during lockdown restrictions. The change in frequency and duration of dog walking was associated with the number of adults in the household, with those with more adults less likely to report reduced walking frequency and duration. This suggests that, in many such households, dog walking may have been shared amongst adult occupants in order to maintain dog walking frequency and duration, while adhering to restrictions on outdoor exercise. This finding appears to contrast with previous results, obtained in the absence of COVID-19, that found a negative association between daily dog walking and the number of household occupants [25]. While that study did not distinguish between adults and children and focused on the outcome of daily dog walking, rather than reduction in dog walking frequency, our findings suggest that the adult composition of a household influenced the impact of lockdown on dogs’ experience of walking. The availability of alternative dog walkers, who did not usually walk the dog, also decreased the odds of reduced frequency, but not duration, of walking. Whilst these people could have included household residents, this finding was independent of the number of adults in the household. Thus, the ability to draw upon support networks, both from within and external to a household, appears to be an effective way for many people to maintain pre-lockdown dog walking frequency levels. However, as there was no such impact on duration of walks, this effect was potentially for relatively short duration functional walks [29] which were not effective in maintaining pre-lockdown walk duration levels, although again the walk duration response categories available may have been insufficiently sensitive to more clearly detect changes in duration.

Certain characteristics of the local area were associated with reduction in walk frequency and duration. Accessibility of off lead walking areas were associated with the frequency and duration of dog walks. Most notably, owners who required transportation to access off lead walking areas were more likely to report reduced walk frequency and duration. Again, this result was anticipated due to government recommendations that people do not travel for exercise and, in terms of impacts on duration, may indicate that those people who usually travel in order to undertake longer recreational walks resorted to functional walks within walking distance of their home [29]. More general features of the local area, in terms of its urban–rural characteristics were also independently associated with walk duration and frequency, with those living in rural/remote areas having significantly lower odds of reduced dog walking frequency, when compared with those living in a village/small town, suburban or city/urban areas. We speculate that this finding may be, in part, associated with different perceptions between areas regarding the risk of enforcement of rules and of encountering other people and/or dogs, with people in more densely populated areas more likely to alter their behaviour due to these concerns.

The age of both the respondent and the dog were associated with the odds of reduced walk frequency, but not duration. Dogs owned by respondents in the younger age categories were more likely to have reduced frequency of dog walking compared to the reference category of 65–74 years, and this effect was greatest for the youngest two categories, 18–24 years and 25–34 years. The reason for this association is unknown but is consistent with a previous study that found that dogs living in households comprising people over 60 years of age were less likely to be walked on a daily basis [25]; hence our result may be related to a lower baseline dog walking frequency within the older age groups which resulted in less scope for change. Reduction in walk frequency was also most evident in those age groups of dogs that may be expected to have been walked the most prior to lockdown; juveniles and young adults. This finding is intuitive as lockdown regulations restricting people to exercising outdoors once a day would be less likely to impact dogs that were previously walked once a day, than dogs that were walked more frequently. However, the authors are not aware of any published literature that reports an association between dog age and frequency or duration of walking. Although data on breed of dog were collected, the variable of dog breed was not included in the multivariable analyses for two reasons. Firstly, the variable of breed had a very large number of categories due to the number of pure breeds represented within our dataset, the presence of crossbreeds and dogs of unknown breeding. Secondly, we were interested in changes occurring during lockdown compared to pre-lockdown, and we hypothesised the direction and extent of these changes would not be impacted by breed.

The impact of COVID-19 restrictions on walk frequency and duration suggest that opportunities for physical health benefits to people and dogs were often reduced at this time. As many other opportunities for physical activity were also curtailed during lockdown (e.g., sport and gyms for people and sporting activities for dogs) the impact on physical wellbeing for both species were likely to have been substantially affected. The potential for a decrease in physical activity (PA) amongst people during the COVID-19 pandemic has been recognised [30] and advice published regarding how best to achieve recommended levels of PA during these unprecedented times [31,32,33]. Similarly, for dogs, the physical health benefits of walking are well recognised, for example to reduce the risk of obesity [34,35].

In addition to a reduction in quantity of walks, the qualities of these walks also changed. Although around one in five dogs were not walked off lead before or during lockdown—similar to the 14.5% reported in a previous a study of UK dogs [25]—the proportion of dogs that were walked on lead (particularly short leads) increased with lockdown and, where dogs were walked off lead, they were more likely to be walked to heel. These changes could have important implications for opportunities for environmental enrichment [36,37] and social interaction with people and dogs [38]. Dogs’ ability to engage in exploratory and sniffing behaviour is reduced when on lead compared to off lead during walks [38] and thus it is likely that the increased use of leads during lockdown reduced the opportunities for dogs to investigate their olfactory environment. This is a concern, as dogs have highly developed olfactory ability [39] and scent is a key mode of communication for dogs [40], with olfactory enrichment shown to be beneficial to dogs in a rescue shelter [41] and to improve dogs’ mood through cognitive stimulation [42]. Furthermore, the effects of changes in walking could lead to dogs experiencing frustration in the short-term [43] and may potentially increase the risk of over-enthusiastic interactions when dog–dog interactions take place again as restrictions are eased. Reduced interaction with other dogs whilst walking during lockdown (Table 3) might be assumed to have resulted in more pleasurable and relaxing dog-walking experiences for owners of dogs who are reactive to other dogs. In contrast, we speculate that owners of dogs who gain pleasure from watching their dogs run freely and interact with other dogs might not have enjoyed dog-walking so much during lockdown if they had been among the group of owners who reported an increased use of leads and/or walking dogs to heel, compared with pre-lockdown.

Evidence indicates that the mental health of people living in the UK deteriorated by late April 2020, compared with pre-COVID-19 levels [44] and researchers have identified an urgent need for global and multidisciplinary study of the mental health effects of the COVID-19 pandemic [45]. Somewhat paradoxically, given the results of this study, suggested strategies to mitigate the negative effects of lockdown restrictions have highlighted the beneficial effects of exercise, and proposals including walking with a dog have been cited [46]. The reductions in dog walking frequency and duration during the lockdown period reported by owners in our study support the results of a recent study which indicated that 21.7% of UK companion animal owners, surveyed between 16 April and 31 May 2020, were worried about their animal(s) because of COVID-19 restrictions to exercise/walks [47]. Hence, owners’ concerns about the welfare of their dogs is an important factor that should not be overlooked.

### 4.2. Changes in Interactions with People and Other Dogs

A particularly notable, though anticipated, effect of lockdown and the Government’s “stay at home” message for many dogs was greatly increased time spent with people. More than two-thirds of dogs were reported to be spending more time with adults and, in households with children, almost 9 in 10 dogs were spending more time with children. Indeed, with almost 90% of respondents reporting that they only left their house for one or more of the reasons classified as essential by the Government, the proportion of dogs not left alone (for more than 5 min) on any given day increased from around 1 in 7 to more than 1 in 2, and the proportion left for 3 or more hours decreased from around 1 in 2 to 1 in 20 with lockdown. Whilst this effect would likely be unevenly spread, with some key workers (including health care staff) working increased hours leading to decreased time spent at home, increased working from home practices and/or furlough and redundancy [5,6,7] are likely to be contributing factors to our finding that dogs were less likely to be left alone in the house and, where this does occur, for shorter time periods during lockdown than previously. There are multiple potential implications of this substantial reduction in time spent alone for affected dogs. Immediate effects of this change could reasonably be hypothesised to include reduced opportunities for dogs to rest and avoid social interaction with family members, including children, potentially increasing the risk of problematic behaviour. Longer-term concerns include the increased risk of SRB (including vocalising, destruction, and toileting when dogs are left alone) when pre-lockdown routines resume, and dogs are left alone at home more frequently and for longer periods. Studies suggest that dogs are affected by the duration of time that they are left alone [48], and that systematic desensitisation (gradual and progressive absence of the owner) is effective in decreasing the severity and frequency of SRB in dogs [49]. Hence, whilst SRB has been estimated to affect up to 50% of pet dogs at some stage of their lives [50], it is reasonable to predict that changes to the lives of owners, as a result of the pandemic, might result in an increased prevalence of SRB. Analysis of follow-up data relating to SRB displayed by the dogs in our dataset will provide unique insight into the role that time left alone, and changes in time left alone (in this case brought about by lockdown restrictions being imposed or lifting) have on SRB.

While many dogs experienced increased time with the people in their households, many also experienced reduced interactions with people and other dogs outside their home. Pre-lockdown such interactions with other dogs were comparable with other research that estimated that approximately 36% of dogs met with 1–2 other dogs on weekdays (28% on weekends), 33% with 3–5 other dogs (35% on weekends), and 21% with 6 or more other dogs (30% on weekends); 9% and 7% interacted with no other dogs on weekdays and weekends, respectively; our calculations from [38]. In contrast, during lockdown, over one-quarter of dogs were reported to have not met with any other dogs during an average day (compared to fewer than 1 in 10 prior to lockdown) and almost 60% of owners reported that their dog spent less time interacting with dogs from outside the household. Furthermore, when meeting other dogs on walks, the proportion of dogs who were permitted by their owner to interact (sniff/play) with those dogs reduced considerably and was influenced by both the familiarity of the other dogs and whether or not the dog was on a lead. Some of this reduction of interaction with other dogs may have occurred because owners prevented interaction with observed dogs, facilitated by increased use of lead walking during lockdown, which has previously been shown to reduce dog–dog interactions [36]. However, during lockdown owners also increasingly chose walking areas in order to avoid locations where they were likely to encounter other dogs and, in consequence, walking locations during lockdown were more likely to be described as “quiet, with few people about” and/or “no dogs, or very rarely any dogs” or “other dogs, but almost always on leads”, when compared with pre-lockdown locations. Choosing walking locations to avoid meeting people, presumably to minimise the risk of COVID-19 transmission, thus also had implications for the number of dogs that owners and their dogs met on walks.

The long-term impact of this period of decreased walking, with fewer opportunities to interact with other dogs remains unknown. To the author’s knowledge, there is no published evidence on whether short-term restrictions on dog interactions may have impacts on their behaviour when interactions can resume. However, it is reasonable to speculate that as more typical levels of dog–dog interactions resume, these could potentially lead to issues in resuming interactions with other dogs, such as increased levels of dog–dog reactivity. Longitudinal data, being collected as part of this study, will provide novel insights into how environmental changes may impact dog behaviour in the long-term. Socialisation opportunities will be particularly relevant to the long-term behavioural development of puppies and warrants further study.

### 4.3. Changes to Enrichment Practices

Enrichment can be used to enhance a dog’s quality of life through social, occupational, physical, cognitive/sensory, and nutritional stimulation [51] and can take place outside the house on walks or within the home. We have shown that lockdown resulted in decreased walking frequency and duration, and thus a decrease in opportunities for enrichment and cognitive stimulation also could have occurred, unless owners compensated by providing dogs with additional enrichment opportunities (play/training and/or provision of toys) within the house and/or garden.

We found evidence for changes in the frequency of providing some forms of enrichment, but not others. There was a marked increase in the proportion of dogs that owners reported playing with, or training, more than once a day (38.1% pre-lockdown, 48.4% during lockdown). In contrast, the frequency with which dogs were given toys to play with varied little in comparison to pre-lockdown levels, although just over half of dogs were given a toy to play with more than once a day, both before and during lockdown. This increase in play/training opportunities reported by owners is encouraging, as training has been noted to provide dogs with important mental stimulation, social stimulation, and behavioural enrichment [as reviewed in [52]. In terms of dog welfare, and providing additional enrichment for dogs during lockdown, the greater tendency to increase the frequency of play/training opportunities involving human interaction, rather than providing toys for the dog to play with alone (or with other dogs), is preferable for two reasons. Firstly, not all dogs will have the company of another household dog and, secondly, although individual differences might exist research has shown that dogs tend to choose dog–human play rather than dog–dog play, and to be more interactive when playing with people than dogs [53]. Furthermore, although few studies have explored the extent to which pet dogs play with toys, studies of dogs kept in research establishments report conflicting results on the extent to which toys and chews are used by dogs from rarely [54] to 24% of their time [55] and dogs housed in rescue shelters may benefit little, if at all, from toys placed in their kennel without accompanying human interaction [56]. Hence, greater increase in social play/training is likely to have resulted in greater benefit to the dogs involved than would have a similar increase in provision of a toy, perhaps something appreciated by the owners involved (either consciously or subconsciously).

Results from generalized linear models suggest that, compared to male owners, female owners had significantly greater odds of reporting that they provided their dog with toys and played games/trained their dogs more frequently during lockdown, compared with pre-lockdown. Similarly, younger owners (aged 18–34 years) had higher odds of increasing the provision of both types of enrichment (playing/training) for their dogs during lockdown. Owners aged 55 years or older had significantly lower odds of increasing the frequency of playing/training with their dogs during lockdown, compared to the reference category of 45–54 year-old owners. The reasons for the associations between owner gender and owner age on provision of both types of enrichment are unclear, but might be related to gender differences in empathy [57] and attitudes towards animals [58]. Alternatively, our finding could be due to unexplored confounding factors. For example, if women and younger owners were more likely to spend more time at home compared to men and older owners.

These models also suggested that both types of enrichment (provision of toys and playing/training with the dog) were more likely to increase in frequency among those owners who reported a reduced total duration of walk time. Interestingly, those owners reporting an increased total duration of walk time were also more likely to report increased frequency of playing/training with their dog. We speculate that owners reporting reduced time spent walking their dog might be intentionally increasing the provision of alternative forms of enrichment in an attempt to compensate for reduced physical and mental stimulation resulting from less walk time. The increased time at home due to the COVID-19 lockdown restrictions may have facilitated some owners to increase both time spent walking their dog and playing/training. The finding that dogs that were walked by “new walkers” (who did not normally walk the dog in early/mid-February 2020) were also more likely to receive increased play/training with the owner is potentially a proxy for those owners who were actively seeking to provide their dog with enrichment levels (walking and/or play/training) comparable with pre-lockdown levels.

During lockdown, dogs living in suburban areas were significantly more likely to have increased frequency of playing/training with their owner than those living in rural/remote areas. Although not reaching significance, there was a trend for dogs living in villages/small towns or city/urban areas to also receive more frequent play/training sessions, when compared with dogs living in rural/remote areas. It could be speculated that dogs living in rural areas could have greater access to open spaces than those in more urban environments, and thus their owners might not have perceived the need to increase the frequency with which they played with/trained their dogs; however, further investigation of the reasons behind this finding is required before conclusions are drawn.

Within our study, puppies (aged 6 months or less) were significantly less likely than the reference category of young adult dogs (1 to <2 years of age) to have increased frequency of play/training time with their owners during lockdown, potentially due to owners of young puppies already often playing/training them pre-lockdown.

### 4.4. Limitations and Future Research

Whilst this nationwide strict lockdown (23 March–12 May 2020) may be a one-off event, numerous locally enhanced lockdown restrictions have subsequently been implemented (such as occurred in Leicester [59]) with additional control measures subsequently imposed nationwide (from 24 September 2020) [60]. More recently, national lockdowns have been introduced on different dates and for different durations for England [61] and Wales [62], whilst national restrictions have been updated in Northern Ireland [63] and Scotland [64]. This pattern of varying lockdown restrictions is likely to continue during the course of the COVID-19 pandemic, or as part of future pandemics. It is also hypothesised that a greater proportion of employees will continue to work from home on a full- or part-time basis [65], following the COVID-19 pandemic, compared with prior to the pandemic, thus impacting the time that dogs are left alone longer-term. Therefore, research into the impact these lockdown measures have had on the wellbeing of dogs is crucial, in order that canine welfare organisations and others can best support dog-owners and dog welfare.

The results of this study are based on data obtained from a large sample of dog owners living in the UK. Most survey questions were optional which resulted in missing data for many questions and the impact of nonresponse bias could not be assessed. Survey completion took approximately 25 min, which is likely to have resulted in a nonrepresentative sample of dog owners, as self-selection is likely to have led to over-representation of owners interested in or motivated towards good welfare for their dog(s). Despite the majority of respondents within our dataset being female (85.1%), responses from 623 male owners were obtained; however, future studies should strive to recruit more male respondents in order to minimise sampling bias related to differential recruitment rates related to owner gender. Despite these limitations, and those associated with self-reported data, the results of this study clearly show the impact on many pet dogs that the UK lockdown regulations (23 March–12 May 2020) had on the daily routine, frequency, duration, and characteristics of walks and interactions with dogs and people.

This study reveals the extent to which specific dog management practices changed during the first stage of lockdown in the UK, as reported by owners (4–12 May 2020), compared with a comparative pre-lockdown period (early/mid-February 2020). Identification of management practices that had changed, likely as a result of government-imposed restrictions, have highlighted areas relating to dog walking, dog–dog and dog–human interactions, including time spent alone, and enrichment that warrant future investigation in terms of the potential long-term impacts on UK pet dogs, including many aspects of behaviour such as SRB and dog–dog reactivity. Dog owners completing the survey reported here were asked for consent to be contacted for follow-up survey completion (to be issued approximately 5 and/or 12 months after the survey detailed here), so that long-term effects of the COVID-19 lockdown on canine health and behaviour can be assessed.

## 5. Conclusions

The vast majority of owners in this study believed their dog’s routine had changed because of lockdown restrictions, and this was evident in many aspects of dogs’ management and exercise. There was a marked reduction in time left alone for many dogs and a concomitant increase in time spent with the adults and children in their households. Many dogs experienced less frequent walks and shorter total duration of daily walks. More of these walks were spent on lead, or at heel, reducing opportunities for interactions with other dogs and for exploratory behaviours, such as sniffing. Reductions in walking were not evenly dispersed and the probability of reduced walking was influenced by dog, owner/household, and environmental factors. However, many dogs experienced increased play/training and more frequent provision of toys, potentially, in part, in compensation for reduced walk frequency/duration. These findings point to the diverse and uneven impacts of the COVID-19 lockdown on dogs in the UK and highlight potential current and future welfare concerns, including the impacts of changes to routine, the potential need for more “alone time”, reduced activity levels and, as household routines return toward “normal”, development or exacerbation of SRB. Although the future need for behavioural restrictions of householders within the UK is uncertain, at the time of writing, curbs on activity are still in place and more far-reaching lockdowns are in place in some areas; thus, the welfare impacts to dogs arising from COVID-19 are ongoing and anticipated to last for many months. Our findings point to the need for engagement with dog owners to mitigate potential welfare impacts and to enhance dog–human relationships to enable mutual benefits in these uncertain times.

## Figures and Tables

**Table 1:** The relative amount of time that the dog was reported to spend with household adults and children, during the last 7 days compared with early/mid-February 2020.

Variable	*n*	%
How much time does (dog name) currently spend with adults in the house, compared with early/mid-February (e.g., because of working longer shifts as a key worker, increased working from home, furlough or redundancy)?		
Currently much more	1965	49.3
Currently a little more	819	20.5
About the same	1160	29.1
Currently a little less	25	0.6
Currently much less	17	0.4
Compared to an average term-time week during early/mid-February 2020, (dog name) has spent...		
Much more time with the child(ren) in the household	405	71.3
A little more time with the child(ren) in the household	86	15.1
The same amount of time with the child(ren) in the household	66	11.6
A little less time with the child(ren) in the household	2	0.4
Much less time the child(ren) in my household	9	1.6

**Table 2:** The number of days/week and the maximum amount of time/day that the dog was reported to have been left alone in the home (during the last 7 days and during a week in early/mid-February 2020).

Variable	Before Lockdown (Early/Mid-February 2020)			During Lockdown (The Last 7 Days)			Relative Change
	*n*	%	Cumulative %	*n*	%	Cumulative %	
Days left alone (for at least 5 min) in a week (*n* = 4276, χ^2^ = 2584.3, df = 7, *p*-value < 0.0001)							
Not at all	622	14.5	14.5	2473	57.8	57.8	4.0
1 day	383	9.0	23.5	837	19.6	77.4	2.2
2 days	461	10.8	34.3	394	9.2	86.6	0.9
3 days	524	12.3	46.5	166	3.9	90.5	0.3
4 days	463	10.8	57.4	75	1.8	92.3	0.2
5 days	901	21.1	78.4	112	2.6	94.9	0.1
6 days	256	6.0	84.4	21	0.5	95.4	0.1
7 days	666	15.6	100.0	198	4.6	100.0	0.3
Maximum duration left alone (*n* = 4272, χ^2^ = 2767.5, df = 6, *p*-value < 0.0001)							
Not at all	336	7.9	7.9	2041	47.8	47.8	6.1
Less than 5 min	33	0.8	8.6	149	3.5	51.3	4.5
5–19 min	84	2.0	10.6	285	6.7	57.9	3.4
20–59 min	299	7.0	17.6	774	18.1	76.1	2.6
1 or more hours but less than 3 h	1454	34.0	51.6	892	18.5	94.6	0.5
3 or more hours but less than 6 h	1691	39.6	91.2	168	3.9	98.5	0.1
6 or more hours	375	8.8	100.0	63	1.5	100.0	0.2

**Table 3:** Daily walk frequency and total walk duration before and during lockdown period, and number of other dogs met (on walks or in an indoor space) during each period.

Variable	Before Lockdown (Early/Mid-February 2020)			During Lockdown (The Last 7 Days)			Relative Change
	*n*	%	Cumulative %	*n*	%	Cumulative %	
Number of walks (*n* = 4408, χ^2^ = 731.07, df = 4, *p*-value < 0.0001)							
Not walked	45	1.0	1.0	107	2.4	2.4	2.4
1 per day	1498	33.4	34.4	2218	49.5	51.9	1.5
2 per day	2012	44.9	79.4	1623	36.2	88.1	0.8
3 per day	678	15.1	94.5	382	8.5	96.7	0.6
4+ per day	247	5.5	100.0	150	3.3	100.0	0.6
Total duration of walks (*n* = 4480, χ^2^ = 70.16, df = 4, *p*-value < 0.0001)							
Not walked	42	0.9	1.0	111	2.4	2.4	2.6
<30 min per day	349	7.7	8.6	313	6.9	9.4	0.9
30–60 min per day	1788	39.6	48.2	1861	41.1	50.4	1.0
1–2 h per day	1733	38.3	86.6	1730	38.2	88.6	1.0
2+ h per day	609	13.5	100.0	518	11.4	100.0	0.9
Number of other dogs met on an average day (on walks or in an indoor space) (*n* = 4297, χ^2^ = 1129.6, df = 3, *p*-value < 0.0001)							
None	338	8.6	8.6	1132	26.3	26.3	3.1
1 or 2 dogs in total	1418	33.0	41.6	1811	42.1	68.5	1.3
3 to 5 dogs in total	1503	35.0	76.5	906	21.2	89.6	0.6
6 or more dogs in total	1009	23.5	100.0	448	10.4	100.0	0.4

**Table 4:** Dog owners’ choice of walk location and use of lead when walking before and during the lockdown period.

Variable	Before Lockdown (Early/Mid-February 2020)		During Lockdown (The Last 7 Days)		Relative Change
	*n*	%	*n*	%	
Choice of walk location (*n* = 4389, χ^2^ = 641.49, df = 2, *p*-value < 0.0001)					
Avoid walking in places where there were likely to be other dogs	943	21.5	1518	34.6	1.6
Go to places with other dogs to allow (my dog) to play	966	22.0	364	8.2	0.4
Choose the walk based on reasons other than the likelihood of meeting other dogs	2480	56.5	2510	57.2	1.0
Use of lead (*n* = 4537, χ^2^ = 391.32, df = 4, *p*-value < 0.0001)					
Not walked	39	0.9	108	2.4	2.8
Short lead	888	19.6	1125	24.5	1.3
Long/flexi-lead	1274	28.1	1454	32.0	1.1
Off lead to heel	377	8.3	420	9.3	1.1
Off lead not to heel	1959	43.2	1430	31.5	0.7

**Table 5:** Comparison of owner-reported selection of walking locations and degree of permitted interactions with other dogs while on walks between periods before and during lockdown.

Variable	Before Lockdown (Early/Mid-February 2020)		During Lockdown (The Last 7 Days)		Relative Change	McNemar’s χ^2^ Statistic	*p*-Value
	*n*	%	*n*	%			
Places dogs were walked (*n* = 4618)							
Rural, out in the countryside	2585	56.7	2112	46.3	0.8	266.17	<0.001
Village or suburban, with some houses and some open space	2050	44.9	2022	44.3	1.0	1.25	0.3
Urban, with lots of houses	782	17.1	749	16.4	1.0	2.55	0.1
Busy, with lots of people about	461	10.1	466	10.2	1.0	0.02	0.9
Quiet, with few people about	1692	37.1	1558	34.2	0.9	18.58	<0.001
No dogs, or very rarely any dogs	590	12.9	694	15.2	1.2	18.68	<0.001
Other dogs, but almost always on leads	955	20.9	1167	25.6	1.2	56.93	<0.001
Other dogs, mostly off the lead	1467	32.2	908	19.9	0.6	344.05	<0.001
N/A—not walked during this time	41	0.9	108	2.4	2.6	52.48	<0.001
Permitted interactions with other dogs (*n* = 2595)							
Unfamiliar dogs, if (my dog) was on the lead	2505	56.9	1329	30.2	0.5	1058.76	<0.001
Familiar dogs, if (my dog) was on the lead	3023	68.6	1934	43.9	0.6	956.95	<0.001
No dogs, if (my dog) was on the lead	690	15.7	1054	23.9	1.5	220.35	<0.001
N/A—(my dog) was not walked on the lead	354	8.0	306	6.9	0.9	14.34	<0.001
Unfamiliar dogs, if (my dog) was off the lead	1700	38.6	1062	24.1	0.6	531.11	<0.001
Familiar dogs, if (my dog) was off the lead	2431	55.2	1624	36.9	0.7	688.90	<0.001
No dogs, if (my dog) was off the lead	344	7.8	494	11.2	1.4	71.62	<0.001
N/A—(my dog) was not walked off the lead	837	19.0	963	21.9	1.2	48.52	<0.001
N/A—(my dog) avoids other dogs	581	13.2	622	14.1	1.1	9.25	0.001

**Table 6:** Frequency with which dogs were reported to spend socialising with other dogs that they do not live with during lockdown, compared to early/mid-February 2020 (*n* = 4182).

Category	Frequency	Percentage
Currently much less	1810	43.3
Currently a little less	671	16.0
The same—no socialising	615	14.7
About the same—some socialising	801	19.2
Currently a little more	209	5.0
Currently much more	76	1.8

**Table 7:** Multivariable models of decreased walk frequency and duration during lockdown, compared with early/mid-February 2020.

Variable	Odds Ratio	Lower Confidence Limit	Upper Confidence Limit	z	*p*-Value
**Model 1: decreased number of walks per day (*n* = 3307)**					
Age of respondent					0.003
18–24 years	1.8	1.1	2.8	2.5	0.01
25–34 years	1.8	1.3	2.3	3.8	<0.001
35–44 years	1.4	1.04	1.9	2.2	0.03
45–54 years	1.4	1.04	1.8	2.2	0.03
55–64 years	1.4	1.1	1.9	2.7	0.006
65–74 years	Reference				
75 years or older	0.8	0.5	1.4	−0.6	0.5
Area					<0.001
Rural/remote	Reference				
Village or small town	1.6	1.3	2.1	3.9	<0.001
Suburban	1.5	1.1	1.9	3.0	0.003
City or urban	1.5	1.1	2.0	2.6	0.01
Home characteristics					0.006
I can walk straight out with (my dog) off lead	Reference				
I can walk out with (my dog) on a lead and can access an area for off lead exercise	1.5	1.1	2.0	2.5	0.01
I need to take (my dog) by car or public transport to access an area for off lead exercise	1.6	1.1	2.4	2.5	0.01
I do not walk (my dog) off lead	1.9	1.4	2.7	3.7	<0.001
Age of dog					0.003
Puppy (≤6 months)	1.4	0.6	3.0	0.7	0.5
Juvenile (7 months to <12 months)	1.9	1.3	2.7	3.2	0.001
Young adult (1 to <2 years)	1.4	1.1	1.8	2.5	0.01
Mature adult (2 to 6 years)	1.1	0.9	1.4	1.3	0.2
Senior adult (7 to 11 years)	Reference				
Geriatric (≥12 years)	0.7	0.5	1.1	−1.5	0.1
Number of adults in household					<0.001
1	Reference				
2	0.6	0.5	0.7	−5.6	<0.001
3	0.4	0.3	0.5	−6.8	<0.001
4+	0.3	0.2	0.4	−7.0	<0.001
New/additional dog walker					0.002
No	Reference				
Yes	0.6	0.4	0.8	−3.4	0.001
**Model 2: Reduction in total duration of walks per day (*n* = 3843)**					
Area					<0.001
Rural/remote	Reference				
Village or small town	1.5	1.2	1.9	3.0	0.003
Suburban	1.7	1.3	2.2	3.7	<0.001
City or urban	1.9	1.4	2.6	4.2	<0.001
Home characteristics					<0.001
I can walk out with (my dog) on a lead and can access an area for off lead exercise	Reference				
I can walk straight out with (my dog) off lead	1.3	0.9	1.8	1.6	0.1
I need to transport (my dog) by car or public transport to access an area for off lead exercise	2.7	1.9	4.0	5.1	<0.0001
I do not walk (my dog) off lead	1.7	1.2	2.4	2.8	0.005
Number of adults in household					<0.0001
1	Reference				
2	0.7	0.6	0.9	−3.2	0.002
3	0.7	0.5	0.9	−2.5	0.01
4+	0.3	0.2	0.5	−5.2	<0.0001

**Table 8:** Frequency with which dogs were given a toy to play with, played games with or participated in training with the owner.

Variable	Before Lockdown (Early/Mid-February 2020)		During Lockdown (The Last 7 Days)		Relative Increase
	*n*	%	*n*	%	
In a week, how often have you, or someone else in your household, played games with or done some training with (your dog)? (*n* = 4101 Stuart–Maxwell χ^2^ = 414.76, df = 7, *p*-value < 0.001)					
Less than once a week	118	2.9	110	2.7	0.9
Once or twice	437	10.7	323	7.9	0.7
3–4 times	690	16.8	500	12.2	0.7
5–6 times	334	8.1	375	9.1	1.1
Once a day	778	19.0	627	15.3	0.8
More than once a day	1570	38.3	1989	48.5	1.3
N/A—(my dog) did not take part in any games or training	146	3.6	161	3.9	1.1
Don’t know/can’t remember	28	0.7	16	0.4	0.6
In a week, how often was (your dog) given a toy to play with? (*n* = 4109, Stuart–Maxwell χ^2^ = 56.042, df = 7, *p*-Value = <0.001)					
Less than once a week	138	3.4	130	3.2	0.9
Once or twice	322	7.8	278	6.8	0.9
3–4 times	357	8.7	328	8.0	0.9
5–6 times	270	6.6	292	7.1	1.1
Once a day	480	11.7	458	11.1	1
More than once a day	2251	54.8	2331	56.7	1
N/A—(my dog) was not given a toy	242	5.9	257	6.3	1.1
Do not know/cannot remember	49	1.2	35	0.9	0.8

**Table 9:** Multivariable models for increased provision of toys and increased play/training with the dog during lockdown, compared with early/mid-February 2020.

Variable	Odds Ratio	Lower Confidence Limit	Upper Confidence Limit	z	*p*-Value
Increased play/training (*n* = 3192)					
Area					0.006
Rural/remote	Reference				
Village or small town	1.3	0.96	1.6	1.6	0.1
Suburban	1.6	1.2	2.1	3.3	0.001
City or urban	1.3	0.9	1.8	1.5	0.1
Respondent gender					0.004
Male	Reference				
Female	1.5	1.1	2.0	2.7	0.006
Respondent age					<0.001
18–24 years	1.8	1.2	2.7	2.8	0.004
25–34 years	1.6	1.3	2.1	3.9	<0.001
35–44 years	1.4	1.1	1.8	2.5	0.01
45–54 years	Reference				
55–64 years	0.7	0.5	0.8	−3.3	0.001
65–74 years	0.6	0.4	0.8	−3.5	<0.001
75 years or older	0.1	0.02	0.3	−3.3	0.001
Dog age					0.04
Puppy (≤6 months)	0.2	0.1	0.7	−2.2	0.03
Juvenile (7 months to <12 months)	1.0	0.6	1.5	0.1	0.9
Young adult (1 to <2 years)	Reference				
Mature adult (2–6 years)	0.9	0.7	1.1	−1.0	0.3
Senior adult (7–11 years)	0.8	0.6	1.1	−1.6	0.1
Geriatric (≥12 years)	0.6	0.4	0.9	−2.2	0.03
Total duration of walks per day					<0.001
Reduced	1.5	1.3	1.9	4.1	<0.001
No change	Reference				
Increased	2.4	1.9	2.9	8.0	<0.001
New/additional dog walker					<0.001
No	Reference				
Yes	1.6	1.2	2.1	3.4	0.001
Increased provision of toys (*n* = 3783)					
Respondent gender					0.005
Male	Reference				
Female	3.0	1.3	8.5	2.4	0.02
Respondent age					0.05
18–24 years	2.9	1.2	6.7	2.4	0.01
25–34 years	2.1	1.1	4.0	2.3	0.02
35–44 years	2.9	1.1	3.7	2.2	0.03
45–54 years	1.6	0.9	3.0	1.6	0.1
55–64 years	Reference				
65–74 years	1.0	0.4	2.2	−0.1	0.9
75 years or older	0.6	0.03	2.9	−0.5	0.6
Total duration of walks per day					0.005
Reduced	2.1	1.3	3.2	3.2	0.001
No change	Reference				
Increased	1.4	0.8	2.3	1.3	0.2

## Data Availability

The data presented in this study are available on request from the corresponding authors. The data are not publicly available due to ethical approval of participant informed consent that included survey respondents being informed that we will remove all personally identifiable information before sharing data with Universities and/or research institutions.

## Supplementary Materials

The following are available online at https://www.mdpi.com/2076-2615/11/1/5/s1 Table S1: Demographic information about respondents their dogs, and household, Table S2: Isolation status of questionnaire respondent and other adult household members.
