# Supplementary material for: Impact of the First COVID-19 Lockdown on Management of Pet Dogs in the UK
Source: Animals (Basel). 2020 Dec 22;11(1):5. doi: 10.3390/ani11010005 (PMC7822167; doi:10.3390/ani11010005)
Supplement: Supplementary file 1 [file animals-11-00005-s001.pdf]

Table S1: Additional demographic information about responders, their dogs and household

	Number	%
**Respondent age group (n=4006)**		
18-24 years	148	3.7
25-34 years	595	14.9
35-44 years	550	13.7
45-54 years	982	24.5
55-64 years	1017	25.4
65-74 years	590	14.7
75-84 years †	112	2.8
≥85 years †	12	0.3
**Number of dogs in household (n=4598)**		
1	3041	66.1
2	1068	23.2
3	310	6.7
4†	94	2.0
5†	36	0.8
6†	23	0.5
7†	13	0.3
8†	6	0.1
9†	2	0.04
10†	2	0.04
>10†	3	0.1
**Age category of dog (n=3945)**		
≤6 months	33	0.8
Juvenile	166	4.2
Young Adult	610	15.5
Mature Adult	2033	51.5
Senior Adult	869	22.0
Geriatric	234	5.9

* categories not included in multivariable models due to low numbers

† these categories combined for multivariable analysis

Table S2: Isolation status of questionnaire respondent and other adult household members.

Person	Status	Yes	Percent yes
Respondent (n=4390)			
	'self-isolating', so do not leave the house/garden at all	171	3.9
	leave the house for one or more of the currently recommended essential reasons (including shopping, medical appointments, exercise including dog walking, caring for vulnerable people)	3569	81.3
	leave the house to travel to work	650	14.8
1 other adult in house (n=2512)			
	They are 'self-isolating', so do not leave the house/garden at all	179	7.1
	They leave the house for one or more of the currently recommended essential reasons (including shopping, medical appointments, exercise including dog walking, caring for vulnerable people	1814	72.2
	They leave the house to travel to work	519	20.7
>1 other adult in house (n=1159)			
	One or more adults are 'self-isolating', so do not leave the house/garden at all	85	7.3
	All other adults are 'self-isolating', so do not leave the house/garden at all	12	1.0
	Some leave the house for one or more of the currently recommended essential reasons (including shopping, medical appointments, exercise including dog walking, caring for vulnerable people)	388	33.5
	All leave the house for one or more of the currently recommended essential reasons (including shopping, medical appointments, exercise including dog walking, caring for vulnerable people	363	31.3
	Some leave the house to travel to work	292	25.2
	All leave the house to travel to work	19	1.6
